# Up-regulated serum levels of soluble CD25 and soluble CD163 in pediatric patients with SARS-CoV-2

**DOI:** 10.1007/s00431-022-04398-8

**Published:** 2022-03-02

**Authors:** Gehan Ahmed Mostafa, Hanan Mohamed Ibrahim, Abeer Al Sayed Shehab, Yasmin Gamal El Gendy, Dina Medhat Mohamed Aly, Ghada Abdel Haleem Shousha

**Affiliations:** 1 Department of Pediatrics, Faculty of Medicine, Ain Shams University, Nasr City, 17 Asem Abd El-Hamid Street off Makram Ebaid, Cairo, Egypt; 2 Department of Clinical Pathology, Faculty of Medicine, Ain Shams University, Cairo, Egypt

**Keywords:** COVID-19, HLH, MIS-C, SARS-CoV-2, sCD25, sCD163

## Abstract

Similar to hemophagocytic lymphohistiocytosis (HLH), some patients with SARS-CoV-2 have cytokine storm. Serum soluble interleukin-2 receptor (sCD25) and soluble CD163 (sCD163) are potential diagnostic biomarkers for HLH that help in guiding its treatment. This study was the first to investigate serum sCD25 and sCD163 levels in SARS-CoV-2. Serum sCD25 and sCD163 were measured by ELISA in 29 patients with SARS-CoV-2, aged between 2 months and 16 years (13 had COVID-19 and 16 had multisystem inflammatory syndrome in children (MIS-C)), in comparison to 30 age- and sex-matched healthy control children and 10 patients with HLH. Levels of these markers were re-measured in 21 patients with SARS-CoV-2 who were followed up 3 months after recovery. Patients with SARS-CoV-2 had significantly higher serum sCD25 and sCD163 than healthy control children (*P* < 0.001). SARS-CoV-2 patients had significantly higher sCD25 than patients with HLH (*P* < 0.05). Serum sCD25 was a good differentiating marker between patients with SARS-CoV-2 and HLH. Although there was a significant decrease of serum sCD25 and sCD163 of the 21 SARS-CoV-2 patients who were followed up, these levels were still significantly higher than the healthy controls levels (*P* < 0.001).

*  Conclusion: *Serum sCD25 and sCD163 levels were up-regulated in SARS-CoV-2 patients. Serum sCD25 was a good differentiating marker between SARS-CoV-2 and HLH. This initial report requires further studies, on large scales, to investigate the relationship between SARS-CoV-2 and both sCD25 and sCD163, including the disease severity and outcome. The therapeutic role of sCD25 and sCD163 antagonists should also be studied in SARS-CoV-2 patients.

**Table:** 

**What is Known:** *• Similar to hemophagocytic lymphohistiocytosis (HLH), some patients with COVID-19 have cytokine storm due to excessive pro-inflammatory host response.* *• *Serum soluble interleukin-2 receptor (sCD25) and soluble CD163 (sCD163) are potential diagnostic biomarkers for HLH. Monitoring of serum sCD25 and sCD163 levels can also help in guiding the treatment.	
**What is New:** *• Serum sCD25 and sCD163 levels are up-regulated in patients with COVID-19, including patients presenting with multisystem inflammatory syndrome in children (MIS-C).* *• Serum sCD25 is a good differentiating marker between SARS-CoV-2 and HLH.*

**What is Known:**

*• Similar to hemophagocytic lymphohistiocytosis (HLH), some patients with COVID-19 have cytokine storm due to excessive pro-inflammatory host response.*

*• *Serum soluble interleukin-2 receptor (sCD25) and soluble CD163 (sCD163) are potential diagnostic biomarkers for HLH. Monitoring of serum sCD25 and sCD163 levels can also help in guiding the treatment.

**What is New:**

*• Serum sCD25 and sCD163 levels are up-regulated in patients with COVID-19, including patients presenting with multisystem inflammatory syndrome in children (MIS-C).*

*• Serum sCD25 is a good differentiating marker between SARS-CoV-2 and HLH.*

## Introduction

The pandemic of coronavirus disease 2019 (COVID-19) is caused by the severe acute respiratory syndrome coronavirus 2 (SARS-CoV-2) [1]. The cytokine storm is induced by the uncontrolled production of the pro-inflammatory mediators. This may induce acute lung injury and adult respiratory distress syndrome in some patients with SARS-CoV-2 [2].

The cytokine storm caused by the novel coronavirus infection, SARS-CoV-2, has significant similarities with the clinical and laboratory findings of hemophagocytic lymphohistiocytosis (HLH) [3]. HLH is a very rapidly progressive systemic inflammatory disease that is characterized by excessive production of cytokines, cytopenias, hyperferritinemia, and many other manifestations. Serum soluble CD25 and sCD163 levels help in the diagnosis of HLH [4]. Monitoring of serum sCD163 and sCD25 levels helps to assess the deterioration of HLH and guide its treatment [5]. Measurement of inflammatory markers assists the clinicians to evaluate the severity, prognosis, and treatment of SARS-CoV-2 [6].

CD25, an IL-2 receptor alpha chain, presents on many cells including activated T cells and regulatory T cells. IL-2–IL-2 receptor (IL-2R) pathway is important in immunity, but it is also involved in the maintenance of self-tolerance. This paradox is further associated with the shedding of IL-2Rα chain, with the production of soluble IL-2R (sCD25). The binding of sCD25 to IL-2 may either enhance or reduce the immune responses depending on the involved target cell in either self-tolerance or immunity. Since levels of sCD25 are increasingly measured in clinical practice, it is important to understand the possible functional impact of IL-2R shedding [7].

CD163, a haptoglobin scavenger receptor, is expressed on macrophages. High CD163 expression in macrophages is a response of the tissues to inflammation [8]. CD163 is subjected to cleavage by the inflammation responsive protease ADAM17 with shedding of the extracellular portion and generation of soluble CD163 (sCD163). This receptor is involved in the clearance of hemoglobin/haptoglobin complexes through macrophage endocytosis. Also, sCD163 has a role in regulating conversion of pro-inflammatory heme to anti-inflammatory heme metabolites; thus, CD163 indirectly contributes to the anti-inflammatory response [9]. In addition, sCD163 is used as a useful parameter that monitors the macrophage activation in inflammatory conditions [10, 11]. The clinical trials of a novel CD25‐directed antibody drug conjugate and the specific targeting of CD163^+^ disease-associated macrophages are underway [12, 13].

This study was the first to investigate serum levels of sCD25 and sCD163 in patients with SARS-CoV-2, including those with multisystem inflammatory syndrome in children (MIS-C), in comparison to patients with HLH and healthy control children. In addition, the levels of these markers were measured in the available patients who were followed up 3 months after recovery.

## Methods

### Study population

This cohort study was conducted on 29 children with confirmed SARS-CoV-2 as defined by the Centre for Disease Control and Prevention (CDC) case definition for COVID-19 [14, 15] over a period of 6 months from the beginning of June 2020 to the end of November 2020. Also, cases fulfilling the criteria for the diagnosis of MIS-C [16] were included. Patients were recruited from the Emergency Department, COVID-19 Isolation Section, and Pediatric ICU at Children’s Hospital, Ain Shams, Cairo, Egypt. We diagnosed COVID-19 from the date of first positive SARS-CoV-2 PCR swab. Based on clinical data and basic laboratory workup results, the degree of the disease severity was identified [14].

#### Exclusion criteria

- Patients with chronic inflammatory diseases, rheumatic diseases, or other autoimmune disorders.
- Patients with malignancies.
- Patients who were receiving corticosteroid therapy or other immune-modulatory drugs and patients who have received intravenous immunoglobulins.

Patients were compared with 30 age- and sex-matched healthy control children and 10 patients with HLH who were diagnosed according to HLH-2004 diagnostic guidelines [17] and were recruited from the Pediatric Allergy, Immunology, and Rheumatology Unit, Children’s Hospital, Ain Shams, Cairo, Egypt. The healthy control children were recruited from the Outpatients Pediatric Clinic, Faculty of Medicine, Ain Shams University, Cairo, Egypt. They had no clinical evidence of a recent infection, previous COVID-19 infection, chronic inflammatory diseases, or rheumatic disorders.

An informed written consent of participation in this study was signed by the parents or legal guardians of the study subjects. This work was approved by the local Ethical Committee of the Faculty of Medicine, Ain Shams University, Cairo, Egypt.

### Study measurements

#### Clinical evaluation of the studied children

This was based on:

- Detailed history taking from caregivers including the history of contact with a COVID-positive patient and the presence of an underlying chronic illness. Parents will be asked about symptoms at disease onset, duration of COVID illness, the presence of fever, respiratory symptoms, gastrointestinal symptoms, anosmia, ageusia, skin rash, or symptoms of organ dysfunction. Therapeutic interventions in the hospital were also recorded.
- Detailed clinical examination was performed for the detection of complexion color, temperature, vital signs, signs of respiratory distress, and skin examination. A complete systemic examination was performed for the detection of organ involvement. Oxygen saturation in room air was measured by pulse oximetry.
- Outcome assessment: All patients were assessed on discharge from the hospital. They were classified according to their fate into patients with complete cure, patients with residual illness, and those patients who unfortunately died.

#### Routine investigations of SARS-CoV-2

Complete blood picture, C-reactive protein (CRP), erythrocyte sedimentation rate (ESR), lactate dehydrogenase enzyme (LDH), liver enzymes, serum creatinine, cardiac enzymes for MIS-C patients, D-dimer, and serum ferritin.

#### Assessment of serum concentrations of sCD25 and sCD163

##### Sampling

Samples were taken on the second day of presentation immediately after being confirmed as COVID-19 or MIS-C. Two-milliliter of whole blood samples were collected from all subjects. After clotting, samples were centrifuged for 20 min at approximately 1000 × g. Sera were separated and stored at −20 °C until time of assay.

##### Principle of the assay

Serum concentrations of sCD25 and sCD163 were measured by using commercially supplied ELISA kits (Wuhan Fine Biotech Co., Ltd.). Principally, a capture antibody is pre-coated onto 96-well plates. Biotin-conjugated antibodies are used as detection antibodies.

##### Method of assay

Samples were thawed at room temperature, vortexed, and diluted as per the manufacturer’s instructions; using the supplied dilution buffer. The control samples were diluted 1:10 and patients’ samples were diluted 1:20. The standards, diluted test samples, and biotin-conjugated detection antibody were added to the wells subsequently and washed with wash buffer after incubation. Addition of HRP-streptavidin was done, and the unbound conjugates were washed away by the use of wash buffer. Visualization of HRP enzymatic reaction was done by using TMB substrates. TMB was catalyzed by using HRP that produces a blue color product which is changed into yellow after adding the acidic stop solution. The yellow color density was proportional to the target amount of the sample captured in plate. The O.D. absorbances of standards and samples were read at 450 nm in a microplate reader. The concentration of target in each sample was calculated by constructing a standard curve via plotting the O.D. at 450 nm of each standard solution on *Y*-axis versus the respective concentration of the standard solution on *X*-axis; then, target concentrations of samples were interpolated from the standard curve. The concentrations from interpolation were multiplied by the dilution factors to obtain the concentrations of targets in samples before dilution.

Clinical evaluation and assessment of the routine inflammatory markers of COVID-19 and serum levels of sCD25 and sCD163 were repeated, for the available 21 SARS-CoV-2 patients, 3 months after recovery.

### Statistical analysis

The results were analyzed by using the commercially available software package (Statview, Abacus Concepts, Inc., Berkley, CA, USA). The parametric data were presented as mean and standard deviation (*SD*). In addition, non-parametric data were presented as median and interquartile range (IQR) which is between the 25th and 75th percentiles. Student’s *t*-test was used to compare the parametric data, while Mann–Whitney test was used to compare the non-parametric data. Spearman’s rho correlation coefficient “*r*” test was used to determine the relationship between the all different variables. For all the tests, a probability (*P*) value of less than 0.05 was considered significant. Patients were considered to have elevated serum sCD25 and sCD163 if their levels were above the calculated highest cutoff values (the 95th percentile of the healthy controls). The highest cutoff value of serum sCD25 calculated from the patients with HLH was measured by ROC curve.

## Results

SARS-CoV-2 patients comprised 16 females and 13 males. Their ages ranged between 2 months and 16 years (mean ± *SD* = 6.5 ± 4.7 years). The control group comprised age- and sex-matched apparently healthy children. They included 18 females and 12 males. Their ages ranged between 2 months and 16 years (mean ± *SD* = 6.5 ± 4.6 years). Patients with HLH comprised 5 females and 5 males. Their ages ranged between 1.5 and 13 years (mean ± *SD* = 6.4 ± 4.6 years).

Thirteen patients had COVID-19 and 16 patients had MIS-C. According to the classification of the degree of the severity of COVID-19, nine patients had moderate COVID-19 and four patients had severe disease, and they were admitted at Pediatric ICU.

Regarding the outcome of the disease, 24 patients recovered (11 had COVID-19 and 13 had MIS-C); one patient, who had COVID-19, died; and 4 patients had residual illness (one had COVID-19 and 3 had MIS-C) in the form of thyroid dysfunction, ophthalmological problems, and neurological dysfunction.

Results of the basic clinical and routine laboratory data of the studied patients are presented in Table 1.

**Table 1:** Basic clinical and laboratory data of the studied children

		**All patients with SARS-CoV-2 (*n* = 29)**	**Patients with COVID-19 (*n* = 13)**	**Patients with MIS-C (*n* = 16)**	**Patients with HLH (*n* = 10)**
**Age (years)**	Range	2 mo–16 yrs	2 mo–16 yrs	1.5 yrs–14 yrs	1.5 yrs–13yrs
	Median (IQR)	7 (7.1)	2 (8.1)	7.5 (4.3)	5 (9.3)
**Sex**	Female	16 (55.2%)	7 (53.8%)	9 (56.3%)	5 (50.0%)
	Male	13 (44.8%)	6 (46.2%)	7 (43.8%)	5(50.0%)
**Outcome**	Recovered	24	11	13	-
	Residual illness	4	1	3	
	Death	1	1	0	
**TLC** (× 10^3^/μL)	Range	2.6–32.0	2.6–26.8	5.3–32.0	1.3–12.6
	Median (IQR)	11.7 (8.8)	10.9 (10.6)	12.3 (6.4)	2.7 (4.8)
**ANC** (× 10^3^/μL)	Range	1.0–22.0	1.0–20.0	2.0–22.0	0.3–8.3
	Median (IQR)	6.7 (7.0)	5.5 (7.0)	7.75 (8.0)	1.6 (3.0)
**ALC** (× 10^3^/μL)	Range	0.3–16.0	0.5–16.0	0.3–6.3	0.5–4.5
	Median (IQR)	2.7 (5.0)	4.4 (6.0)	2.3 (4.0)	1.2 (2.0)
**Hemoglobin** (g/dL)	Range	7.0–14.0	8.0–13.0	7.0–14.0	4.0–12.0
	Median (IQR)	11.0 (2.0)	11.70 (3.0)	11.0 (2.0)	9.25 (3.0)
**Platelets** (× 10^3^/μL)	Range	47–530	114–530	47–395	8–417
	Median (IQR)	203 (202)	203 (248)	187 (151)	162 (248)
**CRP titer** (mg/L)	Range	2.5–423	6.0–171	2.5–423	6.0–229
	Median (IQR)	53 (127)	23 (111.5)	100.5 (178)	31.5(101.8)
**Ferritin** (ng/mL)	Range	33–2066	33–784	56–2066	145–10,325
	Median (IQR)	366 (336)	190 (262)	414.5 (234)	1520(1833)
**ALT** (IU/L)	Range	3.0–238	5.0–238	3.0–163	8.0–258
	Median (IQR)	25 (52)	14 (23)	35.5 (113)	64 (80)
**Creatinine** (mg/dL)	Range	0.3–3.8	0.3–0.9	0.5–3.8	0.3–0.6
	Median (IQR)	0.7 (0.3)	0.5 (0.2)	0.7 (0.2)	0.4 (0.1)
**LDH** (IU/L)	Range	46–1833	179–1833	46–549	243–4986
	Median (IQR)	358 (227)	396 (237)	322.5 (204)	703 (1773)
**D-dimer** (μg/mIF)	Range	0.4–16.5	0.4–16.5	1.4–11	1.7–8.3
	Median (IQR)	2.6 (4.1)	2.3 (4.2)	3.1 (7.5)	3.5 (2.1)
**ESR** (mm/hr)	Range	3.0–45	11–45	3.0–35	5.0–110
	Median (IQR)	20 (21)	25 (27)	16.5 (19)	41 (75)
**CK-T** (IU/L)	Range	-	-	15–278	-
	Median (IQR)			42.5 (140)	
**CK-MB** (U/L)	Range	-	-	10–124	-
	Median (IQR)			20 (15)	
**Troponin I** (ng/mL)	Range	-	-	0.0–1.3	-
	Median (IQR)			0.05 (0.19)	

*ALC* absolute lymphocytic count; *ALT* alanine transaminase; *ANC* absolute neutrophil count; *CK-MB* creatinine kinase-MB; *CK-T* creatinine kinase—total; *COVID-19* coronavirus disease 2019; *CRP* C-reactive protein; *ESR* erythrocyte sedimentation rate; *HLH* hemophagocytic lymphohistiocytosis; *IQR* interquartile range; *LDH* lactate dehydrogenase; *MIS*-*C* multisystem inflammatory syndrome in children; *SARS-CoV-2* severe acute respiratory syndrome coronavirus-2; *TLC* total leucocytic count

**Fig. 1:**
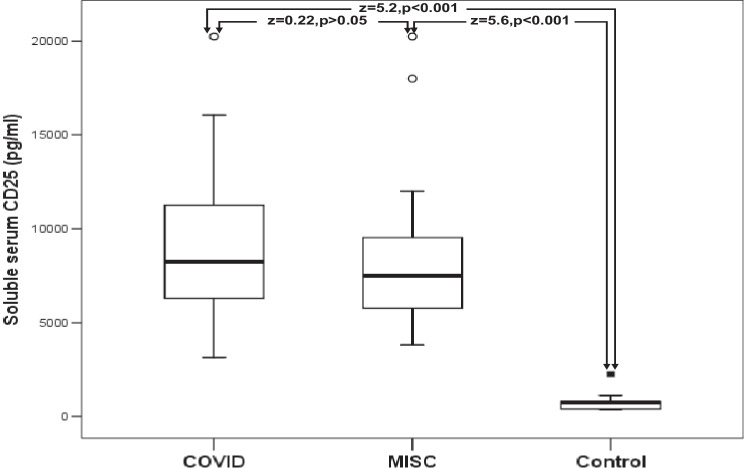
Comparison between patients with SARS-CoV-2 and healthy control children in serum levels of sCD25. The boxes enclose the interquartile ranges (IQR) which are between the 25th and the 75th percentiles. The horizontal line inside the box represents the median, and the whiskers represent the non-outlier or extreme maximum and minimum values. The closed small squares represent extreme values (more than 3 IQR), and small open circles represent the outlier values (between 1.5 and 3 IQR)

**Fig. 2:**
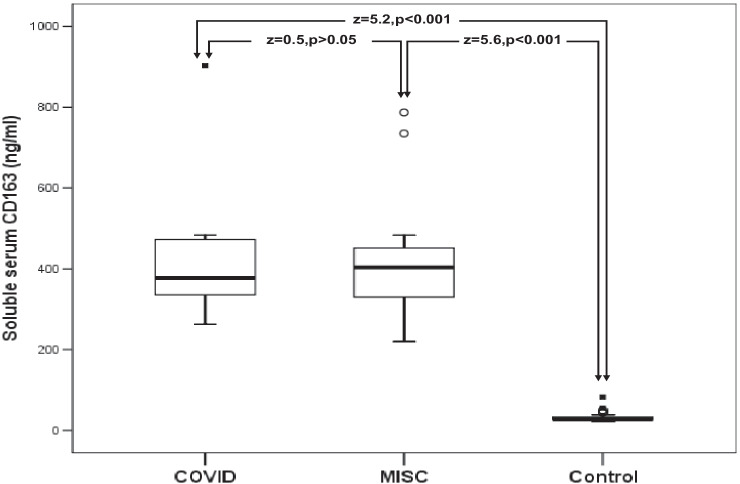
Comparison between patients with SARS-CoV-2 and healthy control children in serum levels of sCD163. The boxes enclose the interquartile ranges (IQR) which are between the 25th and the 75th percentiles. The horizontal line inside the box represents the median, and the whiskers represent the non-outlier or extreme maximum and minimum values. The closed small squares represent extreme values (more than 3 IQR), and small open circles represent the outlier values (between 1.5 and 3 IQR)

All patients with SARS-CoV-2, patients with COVID-19, patients with MIS-C, and patients with HLH had significantly higher values of sCD25 and sCD163 than healthy control children (Table 2; Figs. 1 and 2).

**Table 2:** Comparison between the studied patients and the healthy control children in serum levels of sCD25 and sCD163

**The studied children**	**sCD25(pg/L)** **Range** **Median (IQR)**	**Z-value** **(*P*-value)**	**sCD163(ng/L)** **Range** **Median (IQR)**	***Z*-value** **(*P*-value)**
**All patients with SARS-CoV-2 (*n* = 29)** **Healthy controls (*n* = 30)**	3150–20,250 8250 (4725)	6.624 (<0.001)	220–903 399 (136)	6.632 (<0.001)
	375–2250 750 (390)		22–83 27.5 (7)	
**Patients with COVID-19 (*n* = 13)** **Healthy controls (*n* = 30)**	3150–20,250 8250 (5475)	5.211 (<0.001)	263–903 378 (142)	5.229 (<0.001)
	375–2250 750 (390)		22–83 27.5 (7)	
**Patients with MIS**-**C (*n* = 16)** **Healthy controls (*n* = 30)**	3810–20,250 7500 (4275) 375–2250 750 (390)	5.582 (<0.001)	220–787 404 (134) 22–83 27.5 (7)	5.599 (<0.001)
**Patients with HLH (*n* = 10)** **Healthy controls (*n* = 30)**	2416–12,000 3862 (4911) 375–2250 750 (390)	4.744 (<0.001)	273–1292 844 (923) 22–83 27.5 (7)	4.767 (<0.001)

*COVID-19*: coronavirus disease 2019; *HLH* hemophagocytic lymphohistiocytosis; *MIS*-*C* multisystem inflammatory syndrome in children; *SARS-CoV-2* severe acute respiratory syndrome coronavirus-2

*P* <0.01: highly significant

Patients were considered to have elevated serum sCD25 and sCD163 if their levels were above 1631.25 pg/L and 67.38 ng/L, respectively, which were the calculated highest cutoff values (the 95th percentile of the healthy controls). Increased values of serum sCD25 and sCD163 were found in all patients with SARS-CoV-2 and in all patients with HLH.

According to the pediatric normal reference range for age, elevated CRP and D-dimer values were found in 89.66% and 93.10%, respectively of all patients with SARS-CoV-2. Lymphopenia, neutropenia, neutrophilia, thrombocytopenia, and elevated levels of ESR, ALT, ferritin, and LDH were found in 48.28%, 3.45%, 51.72%, 34.48%, 41.38%, 24.14%, 65.52%, and 75.86%, respectively, of these patients (Table 3).

**Table 3:** Percentage of the abnormalities of the levels of the routine inflammatory markers of SARS-CoV-2, sCD25, and sCD163 in patients with SARS-CoV-2

**Laboratory markers**	**All patients with SARS-CoV-2 (*n* = 29)**	**Patients with COVID-19 (*n* = 13)**	**Patients with MIS-C (*n* = 16)**
**Lymphopenia**	14/29 (48.28%)	5/13 (38.46%)	9/16 (56.25%)
**Neutrophilia**	15/29 (51.72%)	5/13 (38.46%)	10/16 (62.25%)
**Neutropenia**	1/29 (3.45%)	1/13 (7.69%)	0.0%
**Thrombocytopenia**	10/29 (34.48%)	3/13 (23.08%)	7/16 (43.75%)
**Elevated CRP**	26/29 (89.66%)	11/13 (84.62%)	15/16 (93.75%)
**Elevated ferritin**	19/29 (65.52%)	5/13 (38.46%)	14/16 (87.50%)
**Elevated ALT**	7/29 (24.14%)	1/13 (7.69%)	6/16 (37.5%)
**Elevated LDH**	22/29 (75.86%)	11/13 (84.62%)	11/16 (68.75%)
**Elevated D-dimer**	27/29 (93.10%)	11/13 (84.62%)	100%
**Elevated ESR**	12/29 (41.38%)	7/13 (53.85%)	5/16 (31.25%)
**Elevated sCD25**	100%	100%	100%
**Elevated sCD163**	100%	100%	100%

*ALT* alanine transaminase; *COVID-19* coronavirus disease 2019; *CRP* C-reactive protein; *ESR* erythrocyte sedimentation rate; *LDH* lactate dehydrogenase; *MIS*-*C* multisystem inflammatory syndrome in children; *SARS-CoV-2* severe acute respiratory syndrome coronavirus-2

**Fig. 3:**
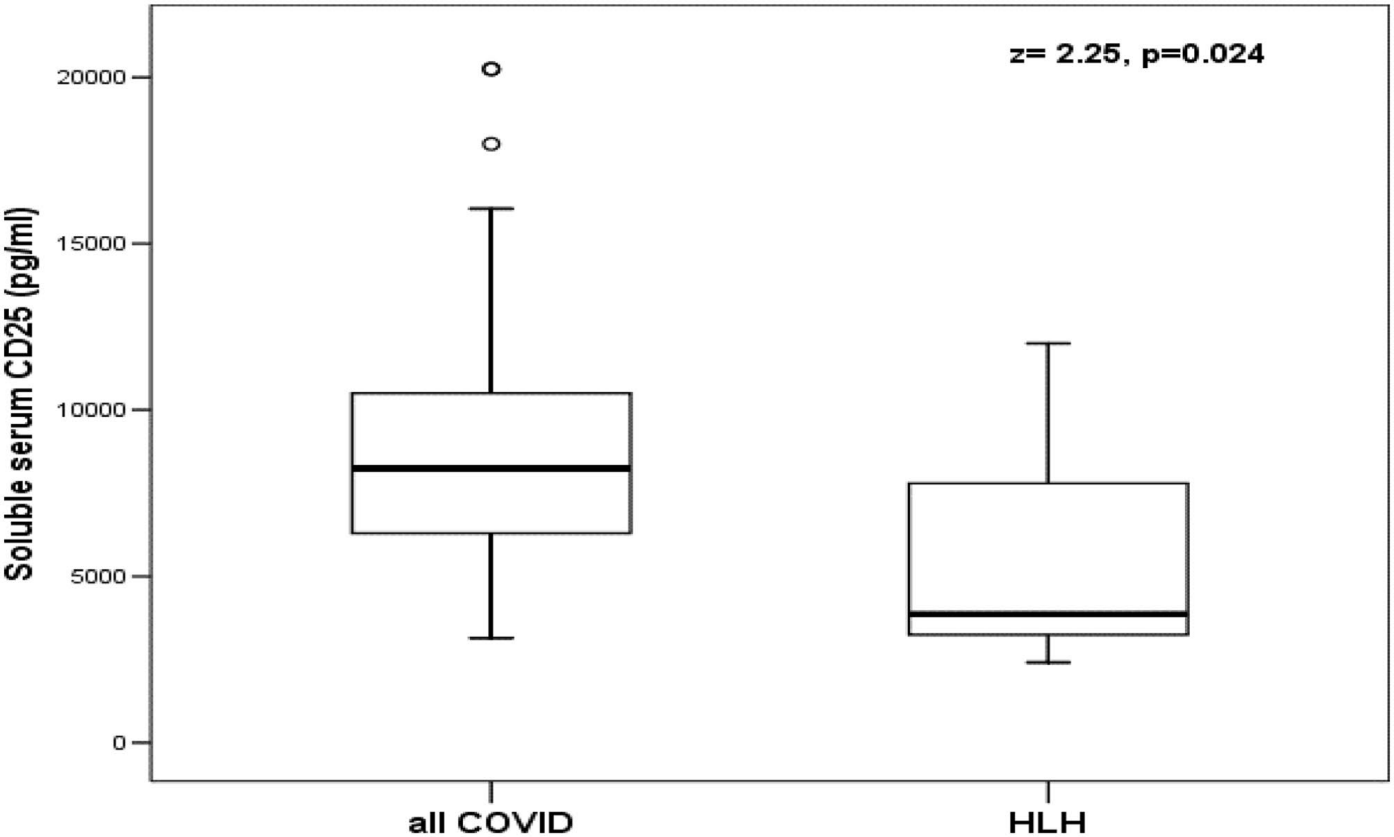
Comparison between all patients with SARS-CoV-2 and patients with HLH in serum levels of sCD25. The boxes enclose the interquartile ranges (IQR) which are between the 25th and the 75th percentiles. The horizontal line inside the box represents the median, and the whiskers represent the non-outlier or extreme maximum and minimum values. The small open circles represent the outlier values (between 1.5 and 3 IQR)

Patients with SARS-CoV-2 had significantly higher values of serum sCD25 than patients with HLH (*P* < 0.05). On the other hand, there were non-significant differences between patients with SARS-CoV-2 and patients with HLH in the levels of serum sCD163 (Table 4; Fig. 3).

**Table 4:** Comparison between patients with SARS-CoV-2 and patients with HLH in the levels of serum sCD25 and sCD163

The studied patients	sCD25 (pg/L) Range Median (IQR)	*Z*-value (*P*-value)	sCD163 (ng/L) Range Median (IQR)	*Z*-value (*P*-value)
**Patients with COVID-19 (*n* = 13)** **Patients with HLH (*n* = 10)**	3150–20,250 8250 (5475)	2.021 (0.042)	263–903 378 (142)	1.304 (0.282)
	2416–12,000 3862 (4911)		273–1292 844 (923)	
**Patients with MIS**-**C (*n* = 16)** **Patients with HLH (*n* = 10)**	3810–20,250 7500 (4275) 2416–12,000 3862 (4911)	1.981 (0.047)	263–903 378 (142) 273–1292 844 (923)	1.582 (0.121)

*COVID-19* coronavirus disease 2019; *HLH* hemophagocytic lymphohistiocytosis; *MIS*-*C* multisystem inflammatory syndrome in children

*P* >0.05: not significant; *P* <0.05: significant

According to the ROC curve, the highest cutoff value of sCD25 calculated from the patients with HLH was 4220 pg/mL. AUC was 0.72 meaning that serum sCD25 was a good differentiating marker between patients with SARS-CoV-2 and patients with HLH at this cutoff value with a sensitivity of 0.89 and 1-specificity of 0.40. Increased values of serum sCD25, above the highest cutoff value, were found in 89.6% of all patients with SARS-CoV-2, 84.6% of patients with COVID-19, and 93.7% of patients with MIS-C.

Patients with HLH had significantly higher values of serum ferritin than patients with SARS-CoV-2 infection (*P* < 0.05).

**Fig. 4:**
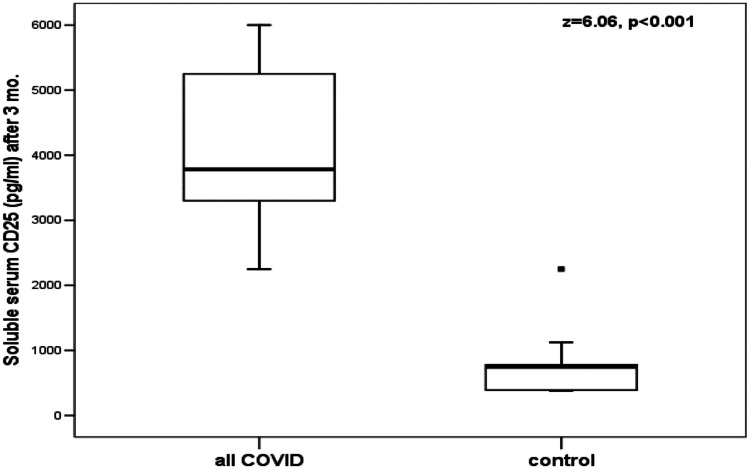
Comparison between serum sCD25 levels of the available 21 patients with SARS-CoV-2 who were followed up 3 months after recovery and the healthy controls. The boxes enclose the interquartile ranges (IQR) which are between the 25th and the 75th percentiles. The horizontal line inside the box represents the median and the whiskers represent the non-outlier or extreme maximum and minimum values. The closed small squares represent extreme values (more than 3 IQR)

**Fig. 5:**
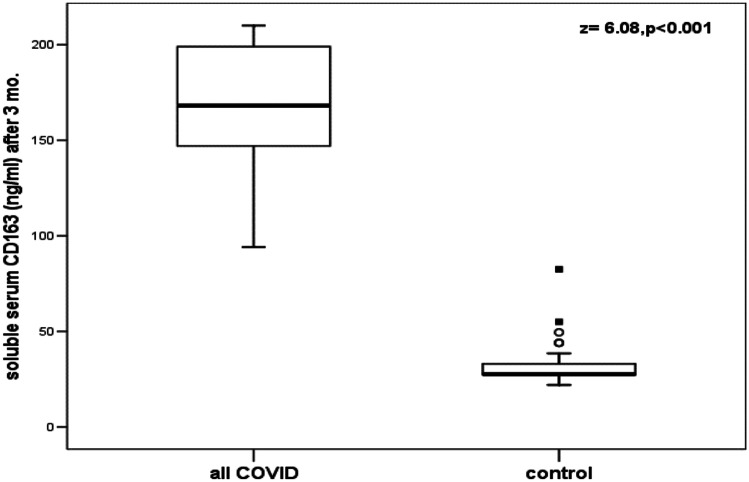
Comparison between serum sCD163 levels of the available 21 patients with SARS-CoV-2 who were followed up 3 months after recovery and the healthy controls. The boxes enclose the interquartile ranges (IQR) which are between the 25th and the 75th percentiles. The horizontal line inside the box represents the median, and the whiskers represent the non-outlier or extreme maximum and minimum values. The closed small squares represent extreme values (more than 3 IQR), and small open circles represent the outlier values (between 1.5 and 3 IQR)

Although there was a significant decrease of serum levels of sCD25 and sCD163 of the available 21 patients with SARS-CoV-2 who were followed up 3 months after recovery, these levels were still significantly higher than the levels of the healthy controls (Table 5; Figs. 4 and 5). Increased values of serum sCD25 and sCD163 above the calculated highest cutoff value of the control group were found in all the 21 patients with SARS-CoV-2 who were followed up. None of these patients had clinical evidence of SARS-CoV-2 with normal values of the routine inflammatory markers of COVID-19.

**Table 5:** Comparison between serum levels of sCD25 and sCD163 of the available 21 patients with SARS-CoV-2, who were followed up, at the onset of the disease and 3 months after recovery

**The studied marker**		**Patients with SARS-CoV-2 who were followed up** **At the onset of the disease (*n* = 21)**	**Patients with SARS-CoV-2 who were followed up** **Three months after recovery (*n* = 21)**	***Z*-value** **(*P*-value)**
**sCD25 (pg/mL)**	**Range** **Median (IQR)**	3810–20,250 8250 (4350)	2250–6000 3780 (1950)	4.016 (<0.001)
**sCD163 (ng/mL)**	**Range** **Median (IQR)**	220–903 399 (100)	94–210 168 (63)	4.019 (<0.001)

*SARS-CoV-2* severe acute respiratory syndrome coronavirus-2

*P* <0.01: highly significant

Significant positive correlations were found between serum levels of soluble CD25 and CD163 in all patients with SARS-CoV-2 (*r* = 0.445, *P* = 0.016) and patients with COVID-19 (*r* = 0.662, *P* = 0.014). In contrast, there were non-significant correlations between serum levels of both sCD25 and sCD163 in patients with MIS-C (*r* = 0.279, *P* = 0.295).

## Discussion

Measurement of inflammatory markers may help in the diagnosis, evaluation of the severity, and monitoring the treatment of SARS-CoV-2 infection [6]. In the current study, patients with SARS-CoV-2 and patients with HLH had significantly higher values of sCD25 and sCD163 than healthy control children. In addition, serum sCD25 and sCD163 were the only inflammatory markers that were elevated in all patients with SARS-CoV-2. CD25 is expressed by T cells during immune activation and its soluble form is released into the bloodstream [18]. CD163 macrophages have a role in hyperferritinemic syndromes. sCD25 and sCD163 were reported to be up-regulated in patients with HLH [5, 11]. Unfortunately, sCD25 and sCD163 levels have not been tested in patients with SARS-CoV-2 [19].

Immune dysfunction, especially cytokine storm and lymphopenia, in some patients with SARS-CoV-2 is a fatal factor for these patients [20, 21]. The elevated levels of inflammatory cytokines in SARS-CoV-2 patients is associated with the decreased number and the increased exhaustion of lymphocytes. In SARS-CoV-2 infection, the mechanism of cytokine-induced lymphopenia is not clear. The elevated levels of inflammatory cytokines in patients with SARS-CoV-2 result in T cell stimulation with a subsequent decrease in their number and increased exhaustion of lymphocytes. [22, 23]. IL-2 is essential for the proliferation, differentiation, and function of T cells [24]. The alpha chain shedding from the T cell surface into the serum (sCD25) is related to the rate of activated T cells proliferation, and this may be the reason behind the up-regulated levels of sCD25 in both COVID-19 and MIS-C patients who had activated T cells proliferation. So, sCD25 is used as a biomarker of the diseases characterized by T cell expansion [25]. Increased serum sCD25 levels predict a decreased cellular response to IL-2. Up-regulated levels of sCD25 may contribute to lymphopenia, which is an indicator of the severity and hospitalization in SARS-CoV-2 infection, through IL-2 signaling inhibition [20]. Serum sCD25 is associated with T cell activation, and it is a marker of disease activity in autoimmune disorders [26]. Elevated sCD25 levels are associated with enhanced antigen-specific Th17 responses [25].

Serum levels of sCD25 are up-regulated in patients with Kawasaki disease who have a systemic inflammatory disease [27]. Some pediatric patients are diagnosed and treated for Kawasaki disease in the setting of confirmed SARS-CoV-2 infection. This may denote the connection between the two diseases and the possible role of sCD25 in SARS-CoV-2 infection [28].

The macrophage is a key cell in the pro- and anti-inflammatory responses. The inflammation microenvironment is rich in CD163 + macrophages [29]. In inflammation, the up-regulated expression of CD163, a macrophage-specific protein, results in the macrophage switch to activated phenotypes. So, a high expression of CD163 in macrophages is a result of tissues response to inflammation and CD163 is a potential biomarker of inflammation and a therapeutic target. Soluble plasma CD163 is the biomarker form of CD163 that results from the increased shedding of CD163 by tumor necrosis factor-α cleaving enzyme during the process of inflammation. sCD163 is up-regulated in many acute and chronic inflammatory disorders [30]. This may be the reason behind the up-regulated levels of sCD163 in both COVID-19 and MIS-C patients who had exaggerated pro-inflammatory host response. Production of both CXCL2 and IL6 and in CD163-deficient macrophages is suppressed. Thus, macrophage CD163 induces IL6 production [31]. In chronic inflammatory diseases, the macrophage is a target for immunotherapy. Recently, drug targeting of the surface marker CD163 expressed in a subpopulation of macrophages is under research. The use of glucocorticoids drug that was coupled to antibodies to CD163 + macrophages in animal models of inflammation revealed a high efficacy with low toxicity [29].

Recent studies in SARS-CoV-2 patients are suggesting a key role of monocytes/macrophages in the pathogenesis of this infection. Also, there is a significant overlap between several features reported in severe SARS-CoV-2 infection and the manifestations included in the HLH-2004 diagnostic criteria [17]. Because HLH is a multi-organ syndrome, the diagnostic approach in a patient with severe SARS-CoV-2 infection in whom HLH is suspected must be carried. In SARS-CoV-2 patients presenting with persistent high fever, progressive pancytopenia, and hepatosplenic involvement, together with hyperferritinemia, hypertriglyceridemia, and hypofibrinogenemia, the suspicion of HLH is high, and the diagnostic workup must be completed with specific immunological and histopathological studies [19]. This study aimed to compare the levels of serum sCD25 and sCD163 of patients with SARS-CoV-2 and patients with HLH.

In the current study, patients with SARS-CoV-2 had significantly higher values of serum sCD25 than patients with HLH. Serum sCD25 was a good differentiating marker between the patients with SARS-CoV-2 and the patients with HLH. Increased values of serum sCD25, above the cutoff value calculated from the patients with HLH, were found in 89.6% of all patients with SARS-CoV-2, 84.6% of patients with COVID-19, and 93.7% of patients with MIS-C. Thus, further studies, on large numbers of patients, are required to calculate the levels of serum sCD25 that could differentiate SARS-CoV-2 from HLH in suspected patients.

A hyper-inflammatory state has been observed in some patients with SARS-CoV2 infection. Elevated serum ferritin levels were reported to be predictors to poor outcomes in patients with SARS-CoV2 infection [32]. Patients with HLH have also elevated serum ferritin levels [33]. The current study revealed elevated serum ferritin levels in patients with SARS-CoV2 and patients with HLH, but these levels were markedly elevated in patients with HLH than patients with SARS-CoV-2. This may denote that the inflammatory state was more excessive in patients with HLH than the studied patients with moderate and severe SARS-CoV2 infection.

In the present study, although there was a significant decrease of serum levels of sCD25 and sCD163 of the 21 patients with SARS-CoV-2 who were followed up 3 months after recovery, these levels were still significantly higher than the levels of the healthy controls. Increased values of serum sCD25 and sCD163 above the calculated highest cutoff value of the control group were found in all the 21 patients with SARS-CoV-2 who were followed up. None of these patients had abnormalities of the levels of the routine inflammatory markers of SARS-CoV-2 infection.

Interestingly, the presence of some CD25 susceptibility alleles has been correlated with both the increased serum levels of sCD25 and the susceptibility to some T cell–driven autoimmune diseases [25, 34–36]. This may explain why serum sCD25 and sCD163 levels in the studied 21 patients with SARS-CoV-2, who were followed up 3 months after recovery, were higher than the levels of the healthy controls in spite of the normal levels of the routine inflammatory markers of SARS-CoV-2. Thus, some individuals who have certain CD25 susceptibility alleles and increased serum levels of sCD25 may be prone to have moderate or severe form of SARS-CoV-2 infection that requires hospitalization or develop MIS-C following SARS-CoV-2 infection. Another explanation may be the occurrence of an immunological scar left by SARS-CoV-2 on the cellular immunity after recovery from the disease as a long-term impact of SARS-CoV-2 infection on the immune system even months after the recovery from disease [37]. Further studies are required to investigate serum levels of sCD25 and sCD163 in patients with SARS-CoV-2 after longer follow-up time after recovery from the disease.

There were significant positive correlations between serum levels of sCD25 and sCD163 in patients with SARS-CoV-2. This may denote that the activated T cell proliferation, which is indicated by the up-regulated levels of sCD25, is associated with macrophage activation, which is indicated by the up-regulated levels of sCD163 in patients with SARS-CoV-2.

Limitation of this study is the small number of the studied children, so we could not investigate the relationship between sCD25 and sCD163 and both the severity and outcome of SARS-CoV-2.

## Conclusion

Serum sCD25 and sCD163 levels were up-regulated in patients with SARS-CoV-2. Serum sCD25 was a good differentiating marker between SARS-CoV-2 and HLH. This initial report requires further studies, on large scales and for longer follow-up periods, to investigate the relationship between sCD25 and sCD163 and SARS-CoV-2, including its severity and outcome.

## Abbreviations

COVID-19: Coronavirus disease 2019
HLH: Hemophagocytic lymphohistiocytosis
MIS-C: Multisystem inflammatory syndrome in children
SARS-CoV-2: Severe acute respiratory syndrome coronavirus-2
sCD25: Soluble CD25
sCD163: Soluble CD163
